# Nr2f1b control venous specification and angiogenic patterning during zebrafish vascular development

**DOI:** 10.1186/s12929-015-0209-0

**Published:** 2015-11-17

**Authors:** Ru-Fang Li, Ting-Yun Wu, Yu-Zheng Mou, Yi-Shan Wang, Chun-Lin Chen, Chang-Yi Wu

**Affiliations:** Department of Biological Sciences, National Sun Yat-sen University, Kaohsiung, Taiwan; Doctoral Degree Program in Marine Biotechnology, National Sun Yat-sen University and Academia Sinica, Kaohsiung, Taiwan; Department of Biotechnology, Kaohsiung Medical University, Kaohsiung, Taiwan

**Keywords:** Nr2f1b, Angiogenesis, vein and tip cell identity, ISV (intersegmental vessel)

## Abstract

**Background:**

The specification of vein and the patterning of intersegmental vessels (ISV) controlled by transcription factor is not fully characterized. The orphan nuclear receptor Chicken ovalbumin upstream promoter transcription factor II (CoupTFII, a.k.a NR2F2) positively regulates vein identity in mice. In this study, we show that *nr2f1b* is important for vein and tip cell identity during zebrafish development.

**Results:**

Nr2f1b mRNA is expressed in ventral lateral mesoderm at 15S stage and in vessels at 24 hpf consistent with a role in early vascular specification. Morpholino knockdown of *nr2f1b* results in a decrease in both vein cell number and expression of the vein specific marker *flt4* and *mrc1*, suggested its role in venous specification. We also show loss of *nr2f1b* reduced ISV cell number and impairs ISV growth, which is likely due to the impairment of angiogenic cells migration and/or proliferation by time-lapse imaging. Consequently, *nr2f1b* morphants showed pericardial edema and circulation defects. Overexpression of *nr2f1b* under the fli promoter increases the number of venous cells and ISV endothelial cells indicated the function of *nr2f1b* is required and necessary for vascular development. We further showed that *nr2f1b* likely interact with *Notch* signalling. *nr2f1b* expression is increased in *rbpsuh* morphants and DAPT-treatment embryos suggested *nr2f1b* is negatively regulated by Notch activity.

**Conclusions:**

We show *nr2f1b* control venous specification and angiogenic patterning during zebrafish vascular development, which is mediated by Notch signalings.

**Electronic supplementary material:**

The online version of this article (doi:10.1186/s12929-015-0209-0) contains supplementary material, which is available to authorized users.

## Background

Vasculogenesis and angiogenesis are two processes to establish the pattern of blood vessels network in vertebrates [1, 2]. In the developing zebrafish trunk, a stereotypic pattern of vascular development begins with dorsal aorta (DA) and posterior cardinal vein (PCV) formation at the midline by fusion of angioblast progenitors migrating from the lateral posterior mesoderm by the 17 somite (17S) stage [3]. Development of the intersegmental vessels (ISVs) of the trunk begins with an angioblast sprouting from the DA, proliferating and migrating dorsally until it reaches the dorsal aspect of the embryo and connects with adjacent ISV cells to form the dorsal longitudinal anastomotic vessels (DLAVs). The leading cells to migrate from the vessel are called tip cells, are proliferative and show multiple filopodia, while the less proliferative, stationary cells which lumenize behind the tip cell are called stalk cells [4, 5].

Many genes and signal pathways have been identified that regulate the specification and maintenance of arterial identity during vasculogenesis, such as vascular endothelial growth factor (VEGF), nrp1, delta-like 4 (dll4), gridlock, foxc1 and foxc2 (reviewed in [6, 7]), Signalling through the Notch receptor is a major contributor to arterial identity as loss of Notch leads to a decrease in the number of cells expressing arterial markers and an increase in cells expressing venous markers [5]. While a number of signaling molecules have been identified to promote arterial identify, there is less description about transcription factors that promote a venous identity. In mice study, the orphan nuclear receptor NR2F2 is expressed in venous endothelial cells [8–10]. Loss of NR2F2 in mouse results in ectopic expression of arterial markers in the vein with loss of the venous endothelial cell identity and acquisition of arterial phenotypes. Thus, NR2F2 functions as the key regulator of venous identity [9]. Recent studies in zebrafish and xenopus showed that *nr2f2* and SoxF regulated venous differentiation [11, 12].

During the development of intersegmental vessel (ISV) sprouts from the dorsal aorta, angioblasts will specify two cell identities, the migratory tip cell that senses attractive and repulsive through the extension of filopodia, or the stalk cell that lumenizes to form an intersegmental vessels (ISVs) [13–15]. ISV angiogenesis has been shown that regulated by VEGF and Notch-flt4 (Fms-related tyrosine kinase 4) signalings. Knockdown of VEGFR2 disrupt ISV formation and loss of Notch signaling results in a significant increase ISV cells and increase in Flt4 expression [5]. Conversely, loss of Vegfc or Flt4 impaired ISV growth and a decreased number of angioblasts in each ISV [16]. Moreover, activation of Notch signaling also results in stalled ISV growth mid-somite, suggesting that Notch represses the Vegfc-Flt4 signaling cascade [17]. In recent years, many molecules, such as cxcr4, UNC5B, angiomotin, pdgfb and trpc1 have been shown involved in angiogenesis [16, 18–21]. Thus, genetic interaction and coordination contributes to the control of endothelial tip-stalk cell behaviors during angiogenesis. Of those factors, NR2F2 in mice has been shown function in angiogenesis mediated by the upregulation of Angiopoietin-1 in addition to its role in venous differentiation [8]. However, we still have limited knowledge of the role of transcription factors in tip cell specification. Surprisingly, nr2f2, which plays a major role in venous specification in mice, acts only a minor role in zebrafish vascular development based on our study and recent publications ([11, 12] and our unpublished results).

During the vascular development in mouse, NR2F2 acts as a major regulator in venous identity and in angiogenic growth [8, 9]. In addition, Nr2f2 interacts with Prox1 physically to specific lymphatic endothelial fate and promote the formation of lymphatic vessels [22, 23]. Our previous study in zebrafish showed that *nr2f2* in zebrafish plays a minor role in venous identity and ISV growth but functions critically in lymphogenesis, similar to recent reports. On the other hand, the related transcription factor NR2F1 is a critical regulator of CNS and peripheral nervous system development and controls cell differentiation in the inner ear [24–26]. However, a vascular function of NR2F1 has not yet been documented.

In this study, we hypothesized that *nr2f1b* has a critical role in blood vessel formation in zebrafish. We showed that loss of *nr2f1b* reduced venous cells and the expression of vein specific markers. We also showed that *nr2f1b* morphant reduces ISV cells and impairs ISV growth. While overexpression of *nr2f1b*, we observed the increase of vein and ISV cells, suggesting that *nr2f1b* play a role in promoting vein and tip cell identity. We further showed that *nr2f1b* functions in vascular development mediated by Notch signalling.

## Methods

### Zebrafish strains and husbandry

Zebrafish (Danio rerio) wild-type Tupfel Long Fin (TL) or transgenic lines: *Tg(kdrl:eGFP)*^*la116*^, *Tg(kdrl:mCherry)*^*ci5*^, *Tg(gata:dsRed), Tg(fli1a:egfp)*^*y1*^ and *Tg(fli1a:negfp)*^*y7*^ have been described [27–30]. Zebrafish were raised and maintained at the 28.5 °C fish room in a 20 L circulating system with filtered fresh water and aeration under the 14 hr: 10 hr (light: dark) lighting conditions. Zebrafish embryos were raised in E3 embryo media (5 mM NaCl, 0.17 mM KCl, 0.33 mM CaCl_2_, 0.33 mM MgSO_4_ and supplemented with 0.25 mg/L methylene blue) at 28.5 °C according to the Zebrafish Book [31]. Embryo development and stages were measured in hour post-fertilization (hpf). Chorions were removed by incubation in 20 mg/ml pronase (Sigma) and endogenous pigmentation was blocked by adding 0.003 % N-phenylthiourea (PTU; Sigma) to E3 media at 6 hpf. All animal experiments are approved from the National Sun Yat-sen University Animal Care Committee (approval reference #10109)

### Whole-mount in situ hybridization

Whole-mount in situ hybridization was performed as described in [32]. *nr2f1b* probe template was amplified by PCR using primers described in Additional file 1: Table S1 and in vitro transcription using T7 Polymerase (Roche) with DIG-labeled UTP. The *flt4, mrc1, notch3* and *ephrinb2* probes have been described [33–35]. Whole-mount *in situ* hybridization (WISH) was performed as previously described [35, 36]. Briefly, Embryos were fixed in 4 % paraformaldehyde in phosphate buffered saline (PBS), permeabilized in 10 μg/mL Proteinase K, hybridized with DIG-labeled probes, washed, reacted with AP-conjugated anti-Dig antibody (Roche) and then proceeded to react with NBT/BCIP substrate (Roche). The reaction was stopped and embryos were fixed with PFA. Embryos were embedded in 3 % methylcellulose (Sigma) and photographed.

### Imaging

Fixed embryos or live embryos were embedded in 3 % methylcellulose or 1.5 % low melt agarose (Invitrogen) and photographed with a Zeiss Axiocam HRc camera (Carl Zeiss) on a Zeiss Lumar V12 stereomicroscope. Confocal images were collected on a Zeiss LSM510 or LSM700 microscope, and stacked images generated by ImageJ or ZEN 2012 software (Carl Zeiss). The number of cells in the vein and ISVs was determined by counting from individual slices of confocal stacks. The counting area is between 5^th^ and 15^th^ ISVs of the embryo.

For histology, embryos were sectioned at 5-7 μM in JB-4 plastic medium (Polysciences, Warrington, PA) and photographed with a Magnafire camera (Optronics, Galeta, CA). Alternatively, embryos were fixed with Tek OCT freezing medium and cryo-sectioned at 10 μM using a Leica CM3050S cryostat and photographed with an SPOT RT3 camera (DIAGNOSTIC Inc.).

### Morpholino and Tol2 DNA Injections

Morpholinos for *nr2f1b* and *rbpsuh* genes were designed and ordered from Gene-Tools, LLC (Philomath, OR), dissolved in H_2_O to a 2 mM stock and further diluted to the working concentration with 0.5 % phenol red (Sigma). Sequences are listed in Additional file 1: Table S1. Microinjections were performed to manipulate gene expression. MOs or expression vectors were injected into 1-2-cell-stage embryos on a 3 % agar plate. After injection, embryos were cultured in E3 buffer. The Tol2kit was used to generate *nr2f1b* overexpression driven by 0.8kb *fli1a* promoter construct [37]. Approximately 100 pg of plasmid DNA was co-injected with 50 pg of Tol2 mRNA into 1-cell embryos. Success of transient *Tg(fli1:nr2f1b)* overexpressing embryos can be verified by GFP signal expression driven by *cmlc* (cardiomyocyte light chain) promoter from the vector backbone.

### RNA extraction, cDNA synthesis and Quantitative RT-PCR (qPCR)

Total RNA was extracted from embryos at desired developmental stages and purified using the Qiagen RNeasy Mini Kit (Qiagen) according to the manufacturer’s instructions. Complementary DNA (cDNA) was synthesized with Superscript III reverse transcriptase and oligo-dT primer (Invitrogen) according to the manufacturer’s instructions. Quantitative RT-PCR was performed using the DNA Engine Opticon System (MJ Research Inc.) with iQ SYBR Green Supermix (BioRad) or using the LightCycle 96 instrument (Roche Inc.) with SYBR Green I Master (Roche). qPCR primers are listed in Additional file 1: Table S1. Relative gene expression levels were analyzed by the ΔΔ C_t_ method, with elongation factor 1α (EF1α) as a reference gene All reactions were performed as biological triplicates.

### DAPT (N-[N-(3,5-Difluorophenacetyl)-L-alanyl]-S-phenylglycine t-butyl ester) treatment

Embryos were treated with DAPT (Sigma), γ-secretase specific inhibitor to block Notch signalling, 75 μM for the working concentration in E3 medium at 6 hpf. Control embryos were treated with an equivalent concentration 0.3 % of DMSO (Dimethyl sulfoxide, Sigma).

## Results

### *nr2f1b* mRNA is expressed in vessels during zebrafish development

We sought to understand the role of the orphan nuclear receptors in venous angioblast development. Loss of NR2F2 in mouse leads to an almost complete loss of the cardinal vein, however, morpholino knockdown of nr2f2 in zebrafish leads to only a reduction in venous marker expression without obvious defects in vein and ISVs ([11, 12] and our unpublished result). Since nuclear receptors subfamily 2 group f members (NR2F2) in mammalian and vertebrates are highly conserved, we hypothesized that *nr2f1b* in zebrafish might play an important role in vasculature.

**Fig. 1:**
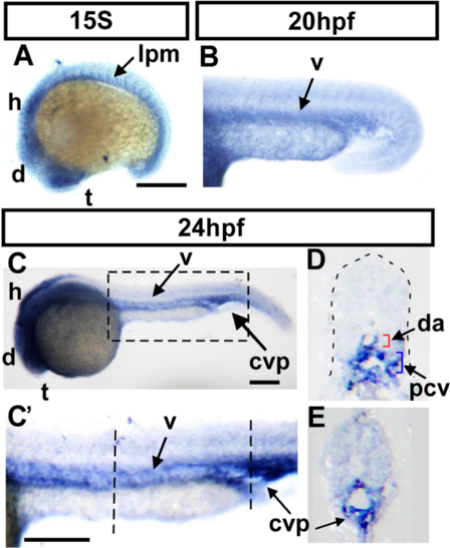
Expression of *nr2f1b* in during zebrafish development. **a** At 15S, *nr2f1b* is expressed in the telencephalon (t), ventral medial diencephalon (d), hindbrain rhombomeres (h) and lateral plate mesoderm (lpm, *arrow*) corresponding in location to the developing vasculature. At 20hpf (~24S), *nr2f1b* is expressed in the vessels (**b**). **c**, **c'** At 24 hpf, *nr2f1b* is expressed in the telencephalon (t), diencephalon (d), hindbrain (h), as well as in vessels (v), and caudal vein plexus (CVP) of the trunk. **c'** is an enlargement of C. **d**, **e** Cross sections of embryos from **c’** show that *nr2f1b* is expressed in dorsal aorta (da), posterior cardinal vein (pcv), and caudal vein plexus (CVP). Scale bars in all figures represent 100 μm.

To examine the role of *nr2f1b* in vascular development, we first analyze the expression of *nr2f1b* by whole-mount in situ hybridization during zebrafish development. At the 15 somite stage (S), *nr2f1b* is expressed in the telencephalon (t), ventral medial diencephalon (d), hindbrain rhombomeres (h) and lateral plate mesoderm (Fig. 1a). The lateral plate mesoderm is the location of vascular precursors. At 20 hpf (~24S), we observed that *nr2f1b* is expressed in the vessels (Fig. 1b). At 24 hpf, *nr2f1b* is expressed in the telencephalon, diencephalon, hindbrain, as well as vessels of the trunk and caudal vein plexus (CVP) (Fig. 1c, c’). Transverse sections of embryos confirm this localization (Fig. 1d, e). The expression of *nr2f1b* in vasculature during embryonic development and suggests that it may play an important role.

### Morpholino knockdown of *nr2f1b* causes vascular defects

**Fig. 2:**
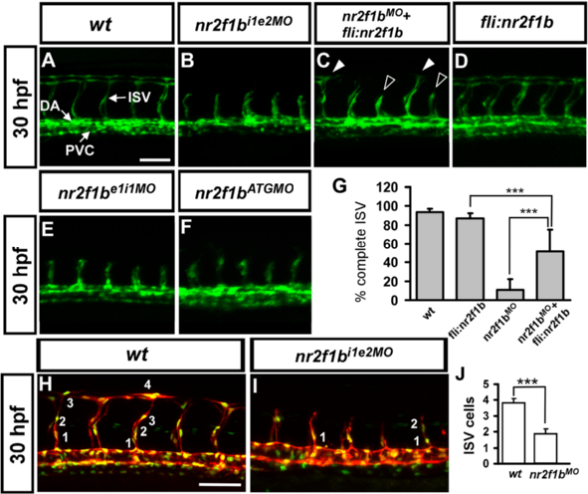
Morpholino knockdown of *nr2f1b* causes defects in vascular development. **a** In uninjected control embryos, the arota (da) and posterior cardinal vein (pcv) have formed by 30 hpf and intersegmental vessels (isv) have reached the DLAV at the dorsal aspect of the embryo. At the same stage ISVs are stalled mid-somite in nr2f1b^i1e2^ (**b**), nr2f1b^e1i1^ (**e**) and nr2f1b^ATG^ (**f**) morphants. Overexpression of nr2f1b has no obvious defect in vasculature (**d**), but rescues the defect of ISV stalling (*solid arrowhead*) as shown in (**c**). **g** Quantification of percentage of completed ISV shows a 40 % increase compare to *nr2f1b* morphants (*** refers to p < 0.0001 by an unpaired student's t-test. Scale bars are 50 μm for (**a**-**f**). **h** and **i** Imaging of endothelial nuclei in green and vessels in red at 30 hpf in wild-type control and nr2f1b MO-treated embryos using Tg(*fli1a:nEGFP)* ^*y7*^ *:(kdrl:mCherry)* ^*ci5*^ double transgenic line. *nr2f1b* morphants showed reduced ISV nuclei numbers (**i**). **j** Quantification of ISV nuclei number in nr2f1b morphants (n = 18) compared to wild-type control (n = 16). ***P < 0.001, Student *t* test.

To identify a functional role of *nr2f1b* in vascular development, we used transgentic fish *Tg(kdrl:eGFP)*^*la116*^ which express GFP in endothelial cells [29] and morpholino-based knockdown technique. We knocked down the expression of *nr2f1b* in embryos by injection of 3 ng of morpholino targeted against the intron 1-exon 2 splice junction (*nr2f1b*^*i1e2*^*MO*), which showed two major vascular phenotypes. First, impairment of ISV growth is observed in *nr2f1b*^*i1e2*^ morphants at 30 hpf, where ISV growth is stalled at mid-somite and ISV pattern is not completed (Fig. 2b) with 89 % of ISVs (n = 400 ISVs from 40 embryos) compared to 7 % of ISVs stalled in uninjected controls (n = 260 ISVs from 26 embryos). The second phenotype we observe is decreased levels of *kdrl:GFP* transgene expression in the posterior cardinal vein (PCV) as compared to uninjected controls at 30 hpf (Fig. 2b). Two additional morpholinos targeting either the translation initiation site (*nr2f1b*^*ATG*^*MO*) or the exon 1-intron 1 splice junction (*nr2f1b*^*e1i1*^*MO*) result in nearly identical phenotypes (Fig. 2e, f), providing evidence for the specificity of the morpholino knockdown. To further confirm the specificity of our morpholino experiments, we performed rescue experiments by overexpression of *nr2f1b* in wild-type and *nr2f1b*^*i1e2*^ morphant embryos. Transient transgenic overexpression of *nr2f1b* in endothelial cells under the *fli1* promoter rescues ISV stalling by 40 % in *nr2f1b* morphants (n = 220 from 22 embryos) compared to injection of *nr2f1b* morpholino alone (Fig. 2c, g), while overexpression of *nr2f1b* in wild-type embryos has no obvious defect on vascular development (Fig. 2d, n = 250 from 25 embryos).

We tested the efficiency and specificity of *nr2f1b*^*i1e2*^ morpholino knockdown. Injection of 1.7 ng or 3 ng of *nr2f1b*^*i1e2*^ morpholino showed dose-dependent disruption of normal splicing of *nr2f1b* as determined by RT-PCR (Additional file 1: Figure S1A, B), suggesting the efficiency of *nr2f1b* knockdown. Sequence comparison of *nr2f1b*^*i1e2*^ morpholino targeting to *nr2f1a* and *nr2f2* showed only 52 % and 28 % identity, respectively. Moreover, injection of *nr2f1b*^*i1e2*^ morpholino greatly decrease the amount of the *nr2f1b* product, but *nr2f1a* and *nr2f2* levels were not decreased compared to uninjected controls as determined by RT-PCR, indicating the specificity of the morpholino knockdown of *nr2f1b* (Additional file 1: Figure S1C, D).

Further, *nr2f1b* morphant phenotypes do not result from morpholino-induced non-specific cell death as there is no significant increase in apoptosis in the trunk of morphants compared to wild type embryos by TUNEL staining (Additional file 1: Figure S2). These results suggested the phenotypes of stalled ISV growth and decreased venous *kdrl* transgene expression are specific to the down-regulation of *nr2f1b* and indicate that *nr2f1b* plays a key role in zebrafish vascular development. To test if loss of *nr2f1b* would decrease cell proliferation, we counted the numbers of endothelial cells per ISV in the *Tg (kdrl:mCherry*^*ci5*^*; fli1a:negfp*^*y7*^*)* embryos, where GFP was expressed in the nucleus of endothelial cells and the mCherry tag in the cytoplasm. Loss of *nr2f1b* showed significantly reduced ISV cell numbers compared to uninjected wild type embryos (1.8 ± 0.8 cells per ISV, n = 107 ISV from 18 embryos of *nr2f1b* morphants and 3.8 ± 0.8 cells, n = 108 ISV from 16 wt embryo, *p* < 0.0001) (Fig. 2 H-J). These data suggest that *nr2f1b* is required for ISV cell growth to contribute to the vascular development, likely by regulation of the proliferation or migration of the cells. Finally, a third phenotype was observed that loss of *nr2f1b* results in pericardial edema, absent parachordal vessels, mispatterned subintestinal vessel plexus and circulation defects at later stages from 48 hpf to 72 hpf (Additional file 1: Figure S3). Since edema and lack of circulation are common secondary consequences of defective blood vessel formation. The circulation defects consistent with the role of *nr2f1b* in vascular development.

### Nr2f1b promotes vein identity

**Fig. 3:**
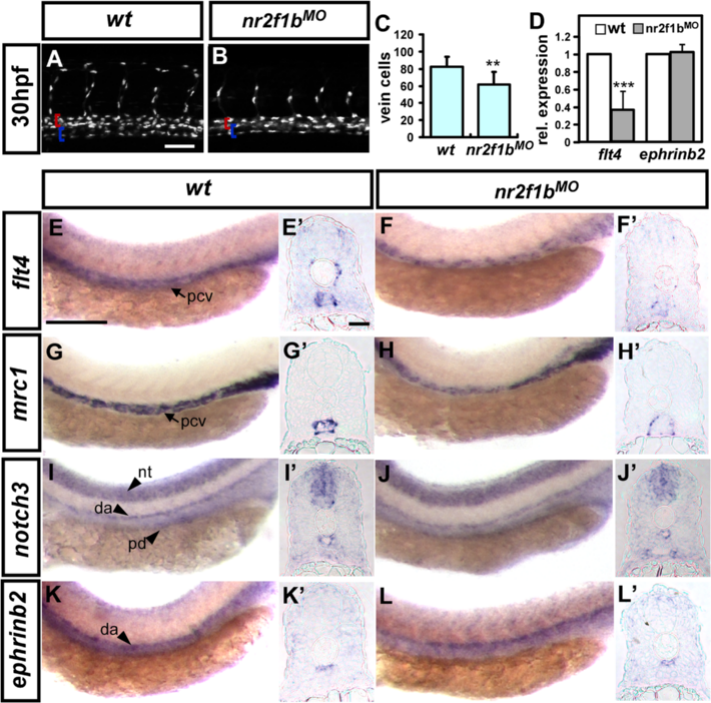
Nr2f1b modulates vein cell number and marker expression. **a**-**c** At 30 hpf, loss of *nr2f1b* function in *Tg(fli1a:negfp)* ^*y7*^ embryos results in reduced vein cell number (**b**, blue bracket) as compared to uninjected wild type controls (**a**). The artery is marked by a red bracket. **c** Quantitative analysis shows a significant reduction in vein cell number in *nr2f1b* morphants. Compared to wild type controls. **e**, **g**, **i**, **k**, expression of the venous markers *flt4* (**f**) and *mrc1* (**g**) is reduced in the trunk of *nr2f1b* morphants at 24 hpf while there is no obvious change in the expression of arterial markers *notch3* (**i**) and *ephrinb2* (**k**). **e’**-**l’** are cross sections of embryos in (**e**-**l**). **d** Quantification by qPCR shows a 60 % reduction in *flt4* expression but no change in *ephrinb2* expression in *nr2f1b* morphants (***refers to p < 0.0001 and **refers to p < 0.001 by an unpaired student's t-test. Scale bars represent 50 μm in **a**, **b**, **e**-**l** and scare bar in cross-sections **e’**-**l’** is 30 μm). Abbreviations: posterior cardinal vein (pcv), dorsal aorta (da), neural tube (nt) and pronephric ducts (pd).

Reduced *kdrl*-transgene expression in the PCV of *nr2f1b* morphant embryos could result from decreased number of cells contributing to the PCV or decreased venous endothelial marker expression of resident endothelial cells. Thus, we analyzed whether the number of endothelial cells is reduced in the PCV in *nr2f1b* morphants by using *Tg(fli1a:neGFP)*^*y7*^ embryos which expressing GFP in endothelial cell nuclei and counting venous cells in the PCV in the region of the yolk extension (i. e. between 5^th^ to 15^th^ ISVs) at 30 hpf. Uninjected control embryos have an average of 82.5 ± 11.5 cells (n = 14 embryos) in this region while *nr2f1b*^*i1e2*^ morphants have a significantly decreased number of cells with an average of 61.8 ± 14.7 cells (n = 12 embryos; p < 0.0001; Fig. 3a-c). Decreased PCV cell number suggests that loss of *nr2f1b* leads to a defect in the specification of venous cells contributing to the PCV but without a fate switch to an arterial fate. Instead, we observed slightly decreased number of cells contributing to the DA in the same region (67.5 ± 7.3 cells in wt and 57.9 ± 8.3 cells, p < 0.01), however, overexpression of *nr2f1b* did not increase the arterial cells (69.1 ± 10.9 cells, n = 10, p = 0.68), suggesting the minor necessary role of *nr2f1b* in aorta differentiation (data not shown).

To determine if loss of *nr2f1b* results in altered expression of arterial and venous markers, we examined the expression *flt4, mrc1, notch3,* and *ephrinb2* by ISH in *nr2f1b* morphants. No obvious differences in the expression of the arterial markers *notch3* and *ephrinb2* were observed in *nr2f1b* morphants compared to controls at 24 hpf by both lateral view (Fig. 3i-l) and cross-section images (Fig. 3i’-l’). Conversely, expression of the venous markers *flt4* and *mrc1* was diminished in *nr2f1b* morphants compared to wild type controls at 24 hpf by both lateral view (Fig. 3e-h) and cross-section images (Fig. 3e’-h’). To determine the extent of decreased marker expression, we quantified *flt4* and *ephrinb2* transcript levels by qPCR and identified a 60 % decrease in *flt4* expression but no change in *ephrinb2* expression in *nr2f1b* morphants (Fig. 3d). These results suggest that the decrease in venous markers expression most likely due to a decreased number of vein cells.

**Fig. 4:**
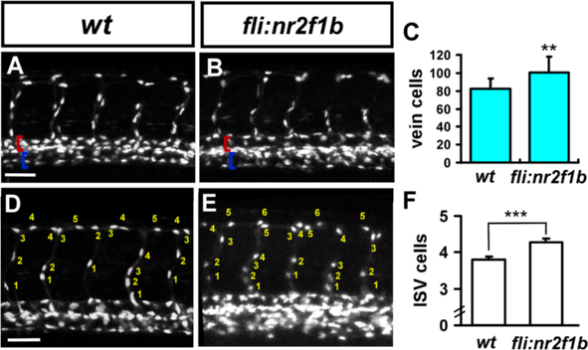
Overexpression of *Nr2f1b* under the fli promoter increases the number of venous cells and endothelial cells per ISV. **a**-**b** The number of vein cells was counted in the region beneath ISV 5-15 from 14 wild type or 10 *Tg(fli1a:nr2f1b)* transient transgenic, overexpressing embryos. The artery is marked by a red bracket, and the vein by a blue bracket. **c** Uninjected control embryos have 82.5 (± 11.5) cells per vein segment while *fli:nr2f1b* over-expressing embryos had 100.3 (± 17.7) cells (p = 0.0068; unpaired t-test). **d**-**f** The number of cells forming each ISV were determined in wild type control (**d**) and *fli1a: nr2f1b* overexpressing embryos (**e**) at 30 hpf. **f** The average cells of wild type control is 3.8 ± 0.7 (ISV n = 86) and the average cells of *fli:nr2f1b* over-expressing embryos is 4.3 ± 0.9 (ISV n = 88). ***refers to *p* < 0.0005 by an unpaired student's t-test and graphic error bars represent 1 SEM.. Scale bars in **a**, **b**, **d**, **e** represent 50 μm.

To test whether excess *nr2f1b* might increase the number of cells contributing to the PCV, we over-expressed *nr2f1b* under control of the *fli1a* promoter. Overexpression of *nr2f1b* in transient transgenic embryos has a slight but significant increase on PCV cell number (100.3 ± 17.7 cells, n = 10) compared to uninjected controls (82.5 ± 11.5 cell; p < 0.01 by student *t-*test) (Fig. 4a-c). Taken all together, the our data suggests that *nr2f1b* has a role in venous endothelial cell specification as loss and over-expression of *nr2f1b* results in decreased and increased numbers of endothelial cells contributing to the PCV, respectively.

### *nr2f1b* modulates the number and migration of angioblasts in intersegmental vessels

**Fig. 5:**
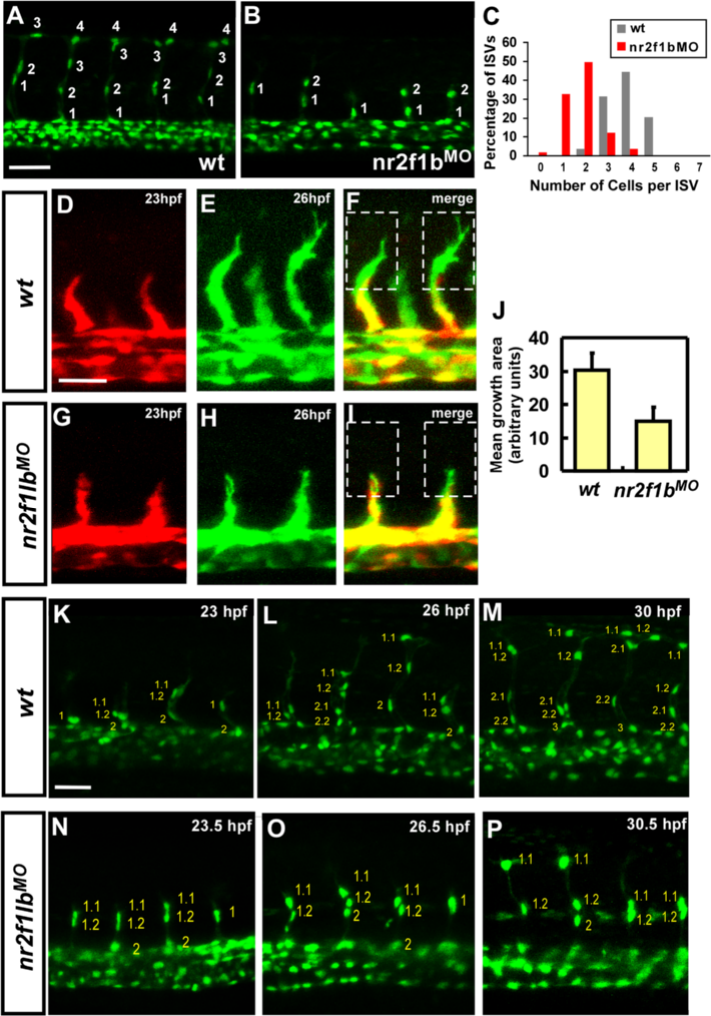
Nr2f1b is required for the growth of intersegmental vessels. The number of cells forming each ISV were counted in wild type control *Tg(fli1a:negfp)* ^*y7*^ (**a**) and *nr2f1b* morphant embryos (**b**) at 30 hpf. **c** Proportional distribution of ISVs containing 1-7 cells in both conditions. **d**-**j** Time-lapse imaging of wild type *Tg(kdrl:eGFP)* ^*la116*^ (**d**-**f**) and *nr2f1b* morphant (**g**-**i**) embryos to examine the extension of ISV tip cell filopodia. Confocal images at 23 hpf (red; **d**, **g**) and 26hpf (green; **e**, **h**) were merged (**f**, **i**). The extension of tip cell was quantitated by pixel intensity and shows reduced extension of tip cell in nr2f1bMO (**j**). **k**-**p** Time-lapse imaging of wild type *Tg(fli1a:nEGFP)* ^*y7*^ (**k**-**m**) and nr2f1b morphant (**n**-**p**). Scale bars represent 50 μm.

The number of cells comprising the trunk intersegmental vessels is regulated by a number of pathways where reduced numbers of cells per ISV can result in ISV growth defect. To determine if a reduced number of cells was present in stalled ISVs, the number of cells per ISV was assessed after knockdown of *nr2f1b* in *Tg(fli1a:negfp)*^*y7*^ or *Tg (kdrl:mCherry*^*ci5*^*; fli1a:negfp*^*y7*^*)* embryos (Fig. 5a-b and Fig. 2h-i). We observed a reduced ISV cells in *nr2f1b* morphants compared to wild-type control (Fig. 2j) and the distribution of ISV cells showed at less cells area in *nr2f1b* MO as compared to uninjected embryos (Fig. 3c). Moreover, endothelial-specific over-expression of *nr2f1b* using the *fli*1 promoter results in an increased average number of cells per ISV (4.3 ± 0.9; n = 88 ISVs) in transient transgenic embryos compared to 3.8 ± 0.7 cells (n = 86 ISVs) in wild type (*p* < 0.0005) (Fig. 4d-f). These data suggest that *nr2f1b* is necessary and sufficient to promote a tip cell identity for ISV growth.

To examine whether migration of ISV angioblasts is deficient, time-lapse imaging was performed in wild type *Tg(kdrl:eGFP)*^*la116*^ and *nr2f1b* knockdown embryos. Superimposition of images of 23hpf embryo (red) on the same embryo at 26hpf (green) shows an average of an over 50 % decreased area of extension/migration of ISVs in *nr2f1b* morphants as compared to wild type embryos (Fig. 5d-j). The decrease in extension could represent decreased protrusive activity of angioblasts, or could result from fewer angioblasts migrating. We therefore examined ISV cell number in *Tg(fli1a:neGFP)*^*y7*^ transgenic embryos on these time-lapse images. We found cells showed slower migration and ISVs that eventually migrate to the DLAV in *nr2f1b* morphants have fewer cells per ISV (Fig. 5k-p) than wild type, suggesting that *nr2f1b* regulates the number of cells in an ISV and the migration of ISV. Together, these data suggest that *nr2f1b* modulates the number and migration of angioblasts during ISV growth.

### Interaction between *nr2f1b* and Notch signaling

**Fig. 6:**
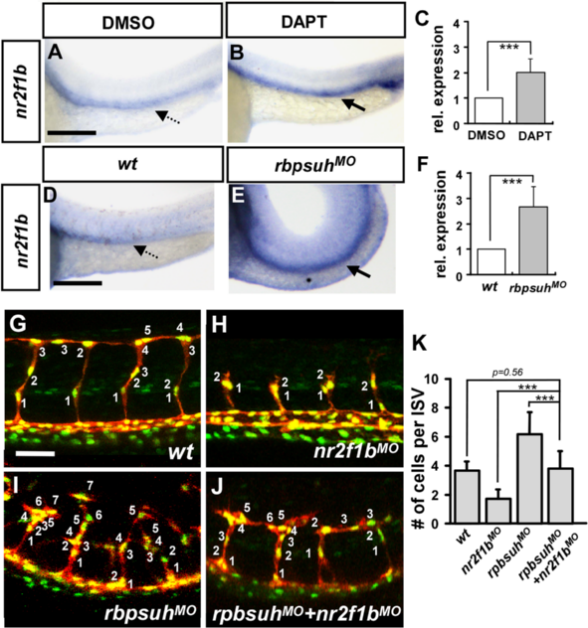
The expression of *Nr2f1b* is upregulated by notch signaling. **a**, **b** *nr2f1b* expression is increased at 24 hpf embryos after treatment with DAPT as compared to DMSO control embryos by in situ hybridization. **d**, **e** *nr2f1b* expression is upregulated in *rbpsuh* morphants at 24 hpf embryos. **c**, **f** Quantification by qPCR showed the increased expression of *nr2f1b* in *rbpsuh* morphants or DAPT treated embryos at 24 hpf significantly. **g**-**j** Representative confocal images showing the number of nuclei per ISV at 30 hpf in **g** wild-type (*wt*) embryos, **h** *nr2f1b* ^*MO*^, **i** *rbpsuh* ^*MO*^, and **j** *rbpsuh* ^*MO*^ with *nr2f1b* ^*MO*^. **k** Quantitation of the average number of cells per ISV in single and double morphants. ***refers to *p* < 0.0005 by an unpaired student's t-test. Scale bars represent 100 μm in **a**, **b**, **d**, **e** and 50 μm in **g**-**j**.

We have demonstrated that knockdown of *nr2f1b* expression by morphlino injection results in ISV stalling at the midline with a decrease in the number of cells per ISV, while over-expression of *nr2f1b* leads to an increased number of cells per ISV. These observations are qualitatively similar to those seen with modulation of Notch signaling where activation of Notch signaling gives ISV stalling at the midline, while loss of *notch* signaling leads to an increased number of cells per ISV (Siekmann 2007). Thus, we sought to determine if Notch regulates *nr2f1b* expression. Notch signaling involves two sequential proteolytic processing events (ADAM protease and γ-secretase) that release the Notch intracellular domain (NICD) into cytoplasm followed by translocation to the nucleus where it interacts with the transcription factor rbpsuh (recombination binding protein/ suppressor of hairless) to activation of target genes. DAPT specifically inhibit γ -secretase involved in the cleavage of NICD and prevents Noch activation. Therefore, to test if *nr2f1b* interacts with Notch signaling, we suppressed Notch signaling by *rbpsuhMO* injection (Fig. 6a-c) or DAPT treatment (Fig. 6d-f). We found *nr2f1b* expression is upregulated when Notch signals were inhibited, with a 2-2.5 fold increase by using in-situ hybridization and qPCR (Fig. 6). These results suggest that nr2f1b might act downstream of Notch signals. We next asked whether nr2f1b and Notch genetically interact to control ISV growth. To test this, we performed a rescue experiment by injecting *nr2f1b* morpholino in an *rbpsuh* morphants (*rbpsuh*^*MO*^). Wild type embryos at 30 hpf have an average of 3.6 ± 0.6 cells per ISV (Fig. 6g, k). *nr2f1b* morphants have an average of 1.7 ± 0.7 cells per ISV, while *rbpsuh* morphants have an average of 6.2 ± 1.6 cells per ISV (Fig. 6h, i, k). Knockdown of *rbpsuh* in combined with *nr2f1b* morpholino injection reduces the number of cells per ISV to wild type levels 3.8 ± 1.2 (Fig. 6j, K; n = 25 ISV from 4 fish; *p = 0.56*, unpaired student t-test). Together, these data suggest *nr2f1b* likely acts downstream of Notch signaling and antagonizes Notch signals to control ISV growth.

## Discussion

### *nr2f1b* expressed in developing vessels functioning in vasculature

In this study, we observed the expression of *nr2f1b* in lateral plate mesoderm at early stage and developing vessels (Fig. 1) corresponding to the location in which primitive angioblasts are developing and acquiring arterial and venous identity. At 24hpf, the expression pattern of *nr2f1b* continues in the vasculature, suggested the role of *nr2f1b* in vascular development. We further have shown that knockdown of *nr2f1b* results in vascular defects, including ISV growth impairment, pericardial edema, less venous cells and results in loss of circulation (Fig. 2 and Additional file 1: Figure S3). In fact, there is no parachorda vessel (PCV, pre-structure of lymphatic duct) formation and defects in caudal vessel plexus (CVP) (data not shown) suggested that the impairment of venous angiogenesis at late developmental stage, which is likely the later effects of loss of *nr2f1b*.

### *nr2f1b* functions in vein identity and the growth of intersegmental vessels

Here, we explored the function of *nr2f1b* modulates vein identity. We showed the decrease of endothelial venous cells and the expression of venous markers *flt4* and *mrc1*; however, there is no obvious change in the expression of arterial markers *notch3* and *ephrinb2* (Fig. 3) although there is a slight decrease of arterial cells. This suggested that *nr2f1b* is not involved in artery-vein fate switching. Thus, the reduction in cell number and the loss of vein marker expression together suggest that *nr2f1b* is necessary for normal vein development. We also showed that overexpression of *nr2f1b* has a slight but significant increase on PCV cell number (Fig. 4). Together, those data suggest the role of *nr2f1b* is necessary and sufficient for vein development.

Intersegmental vessels form from angioblasts sprouting from the dorsal aorta and vein. Stalling of intersegmental vessel growth at the mid somite might therefore either occur through defective proliferation or defective migration of cells. Here, we showed in *nr2f1b* morphants contains less cells compared to in wild-type (Fig. 5). We also showed a significant increase in ISV cell numbers suggesting that *nr2f1b* is necessary and sufficient to promote a proliferation of ISV cells. Further examining the migration of ISV angioblasts in *nr2f1b* knockdown *Tg(fli1a:eGFP)*^*y1*^ and *Tg(fli1a:neGFP)*^*y7*^embryos, we showed that the decrease in extension represent the decreased protrusive activity of angioblasts and fewer angioblasts in migrating (Fig. 5), suggesting that *nr2f1b* regulates the number of cells in an ISV and the migration of ISV. Loss of *nr2f1b* in zebrafish leads to decreased numbers of cells in the posterior cardinal vein and in ISVs, but the ultimate fate of these cells remains unknown. TUNEL analysis suggests that cell death is not increased in the trunk region and we did not observe additional cells contributing to the artery or ISVs. This suggests there may be a lack of proliferation of venous precursors or these cells may adopt a closely related fate in the mesoderm lineage, such as blood.

### Interaction between *nr2f1b*, *nr2f2* and *isl2* in regulating vein identity and ISV growth?

We previous identified *isl2* promotes vein and tip cell identity (paper under revision) and *nr2f2* also plays minor role on that although NR2F2 is a major determinant of venous identity in mouse. In this study, we showed that zebrafish Nr2f1b ortholog to mouse NR2F2 plays a major role in vein and tip cell identity. It has been shown that LIM-homeodimer transcription factor isl1 (an ortholog of Isl2) and CoupTFI physically bind together *in vitro* and *in vivo* to activate transcription [38], suggesting the possible interaction between isl2 and nr2f1b to control vascular development. Thus, whether Nr2f1b/Nr2f2 and Isl2 also physically interact at the protein level to activate target genes and regulate endothelial cell identity remains an interesting avenue to explore in the future. Meanwhile, it would be also intriguing to address if any other signaling molecules in addition to notch, such as vegf, wnt or BMP etc. that interact with nr2f1b and/or isl2. The molecular mechanisms that how *nr2f1b* regulate its targets in vascular development is still unknown and we are currently addressing this question by processing and analyzing genome-wide transcriptome results.

### Does zebrafish Nr2f1b play a conserved role in vasculature similar to nr2f2 in mice?

Swift’s study showed SoxF factors and Notch regulate nr2f2 gene expression during venous differentiation [12] and Aranguren et al showed coupTFII functions in venous and lymphatic development in both zebrafish and Xenopus [11]. However, knockdown nr2f2 in both studies did not see obvious defects in vascular development, but reduction of venous gene expression, suggest nr2f2 control venous differentiation, instead of specification. In this study, we showed that zebrafish Nr2f1b ortholog to mouse Nr2f2 and plays a major role in vein and tip cell identity, which is consistent with the function of nr2f2 in mice [9]. Those data indicate that the conserved vascular function of coupTF family among the vertebrates. Phylogenetic analysis of coupTFs amino acids among the vertebrates suggests that zebrafish nr2f1b and nr2f2 are very closer to mammalian nr2f2 (over 83 % identical). It would be intriguing to address if any functional rescue or compensation between zebrafish nr2f1b and mouse nr2f2.

## Conclusions

In summary, our study demonstrated that *nr2f1b* has a critical role in blood vessel formation in zebrafish. We showed that loss of *nr2f1b* reduced venous cells and the expression of vein specific markers. We also showed that *nr2f1b* morphant reduces ISV cells and impairs ISV growth. While overexpression of *nr2f1b* increase vein and ISV cells, suggesting that *nr2f1b* play a role in promoting vein and tip cell identity. We further showed that *nr2f1b* functions in vascular development in concert with Notch signalling.

## Additional file

Additional file 1:
**Supplementary Methods.** Table S1: Primer/morpholino sequences used in this study. Figure S1: Knockdown efficiency and specificity of *nr2f1b* morpholinos. Figure S2: An increase in non-specific cell death after morpholino injection is not the cause of the observed vascular phenotype. Figure S3. Loss of *nr2f1b* results in pericardial edema, absent parachordal vessels, subintestinal vessels (SIV) mispattern and circulation defects. (DOC 2967 kb)
