# Supplementary material for: Nr2f1b control venous specification and angiogenic patterning during zebrafish vascular development
Source: J Biomed Sci. 2015 Nov 17;22:104. doi: 10.1186/s12929-015-0209-0 (PMC4647328; doi:10.1186/s12929-015-0209-0)
Supplement: Additional file 1: — Supplementary Methods. Table S1: Primer/morpholino sequences used in this study. Figure S1: Knockdown efficiency and specificity of nr2f1b morpholinos. Figure S2: An increase in non-specific cell death after morpholino injection is not the cause of the observed vascular phenotype. Figure S3. Loss of nr2f1b results in pericardial edema, absent parachordal vessels, subintestinal vessels (SIV) mispattern and circulation defects. (DOC 2967 kb) [file 12929_2015_209_MOESM1_ESM.doc]

**Supplementary Methods:**

**Morpholino efficiency**

The efficiency of morpholinos in causing mis-splicing was measured using PCR with primers spanning either side of the targeted exon. For nr2f1b, primers nr2f1b_r2 forward and nr2f1b_pr2 reverse were used, while for EF1α (a loading control), primers EF1α_4F and EF1α_1R (set 1) or primers EF1α_2F and EF1α_2R were used (set 2).

**TUNEL staining**

Embryos were fixed in 4% PFA overnight, dehydrated in MeOH, and stored at -20°C. Embryos were rehydrated in sequential 5 min washes in 75%, 50% and 25% MeOH, and washed 2x in PBT. Embryos were digested in 100µL of 10µg/mL Proteinase K for 20 minutes, and then fixed in 4% PFA for 15 minutes. Embryos were then treated with 3% Hydrogen Peroxide in 0.1% PBT for 1 hour at room temperature and washed with PBT to eliminate endogenous peroxidase. 5 µL TUNEL enzyme solution (Roche) was pre-mixed with 45 µL of TUNEL label solution per sample. This mixture was added to the embryos and incubated in the dark for 3 hours at 37°C. As a negative control, 50 µl of TUNEL label solution was used without enzyme. Embryos were rinsed for three times in PBT to remove unincorporated nucleotides. Embryos were then blocked in 5% Normal Sheep Serum (NSS) in PBT for 2 hours at room temperature, then incubated with peroxidase conjugated anti-Fluorescein antibody diluted 1:2000 (Roche) in 5% NSS in PBT over night at 4°C, washed four times in PBT, and visualized using DAB (Vector Laboratories).

**Table S1: Primer/morpholino sequences used in this study**

| **Conventional PCR primers** | **Sequence** |
| --- | --- |
| nr2f1b^i1e2^ MO efficiency-f | CCAACAGAAACTGCCCGGTG |
| nr2f1b^i1e2^ MO efficiency-r | GGAGCAGCAGTTTGCCGAAG |
| EF1α- 4F | AGCTGATCGTTGGAGTCAAC |
| EF1α- 1R | GGATGATGACCTGAGCGTTG |
| **qPCR primers** | **Sequence** |
| nr2f1b f3 qpcr | AGCCATAGTGCTGTTCACCTCAGA |
| nr2f1b r3 qpcr | ACATACTCCTCCAGGGCACATTGA |
| EF1α_2f | TCAACGCTCAGGTCATCATC |
| EF1α_2r | GATGTGAGCAGTGTGGCAATC |
| flt4_f1 | ACTCGGGTTATTACCGCTGCTTCT |
| flt4_r1 | TGGATGCTCTGGGTCTCGAACAAA |
| ephrinb2_qf | CTGGAACACCACGAACACC |
| ephrinb2_qr | CACACGTGGGCAAACTATGT |
| **Morpholino Name** | **Sequence** |
| nr2f1b^ATG^ MO | ATCTCTCCACGCGCTCACCACCATG |
| nr2f1b^e1i1^ MO | CCCACACAAGATGTACTCACCTTCG |
| nr2f1b^i1e2^ MO | AACCGCTATCAGAACACAGAGAGAT |
| rbpsuh ORF-MO | CAAACTTCCCTGTCACAACAGGCGC |


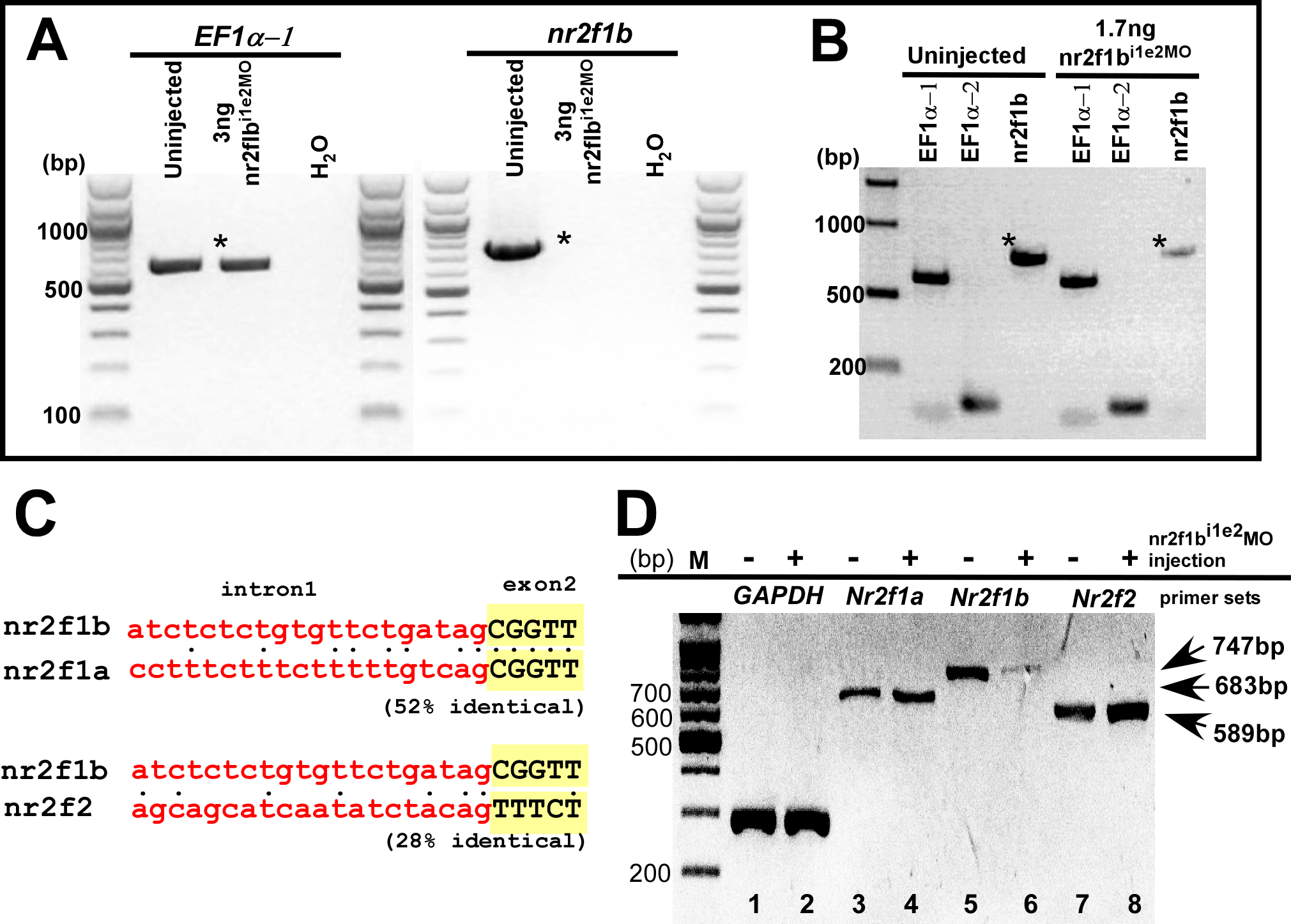


**A**

**Figure S1: Knockdown efficiency and specificity of nr2f1b morpholinos**

(A, B) cDNA from uninjected controls or *nr2f1b* morphants (injected with 3 ng or 1.7ng morpholino) underwent PCR with primers for the different sets of housekeeping control genes (*EF1α-1* or *EF1α-2*), or for *nr2f1b*. In morphants injected with *nr2f1b* ^i1e2^, *EF1α* levels are unchanged while the amount of wild-type *nr2f1b* product is diminished (* in A) or greatly reduced (* in B).

(C, D) Specificity of nr2f1b morpholino targeting: (C) Nr2f1b splicing morpholino sequence design was targeted against the intron1-exon2 splice junction. The sequence comparison to nr2f1a and nr2f2 are only 52% identity and 28% identity, respectively, suggesting the morpholino targeting is nr2f1b-specific. (D) cDNA from uninjected controls (-) or nr2f1b^i1e2MO^ injected embryos (+) underwent PCR with primer sets of the control gene *GAPDH*, *nr2f1a*, *nr2f1b* and *nr2f2.* In *nr2f1b^i1e2MO^* injected morphants, *nr2f1a* (683bp) and *nr2f2* (589bp) levels are not decreased (lane 3-4, 7-8) while the amount of *nr2f1b* product (747bp) is largely decreased (lane 5-6) compared to uninjected controls, indicating the morpholino knockdown of *nr2f1b* is specific.


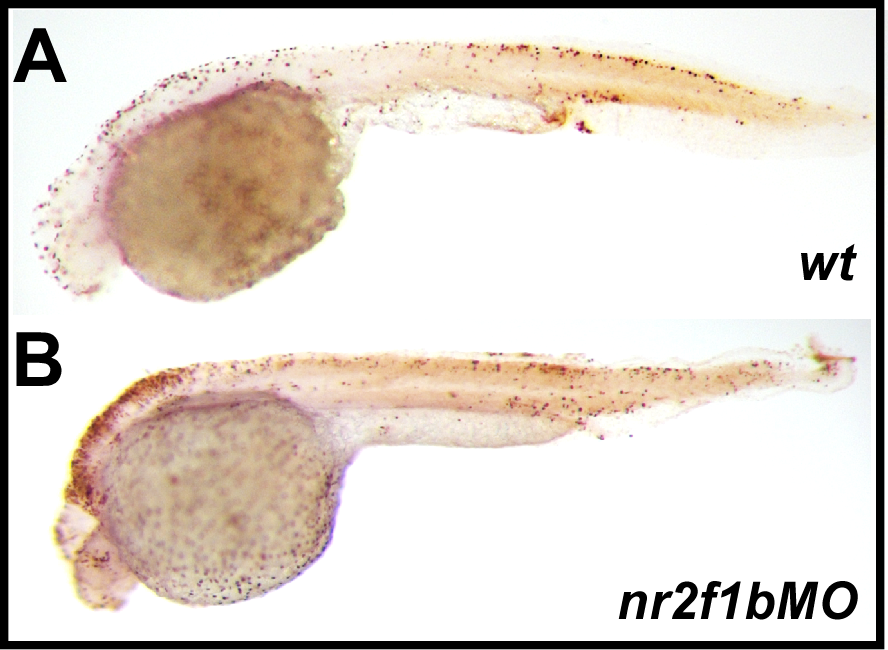


**Figure S2: An increase in non-specific cell death after morpholino injection is not the cause of the observed vascular phenotype**

TUNEL labeling was used to detect apoptotic cells in wild type and *nr2f1b* morphants. Some cell death was observed at the head region, but cell death in vascular regions was not elevated over that observed in wild type controls.


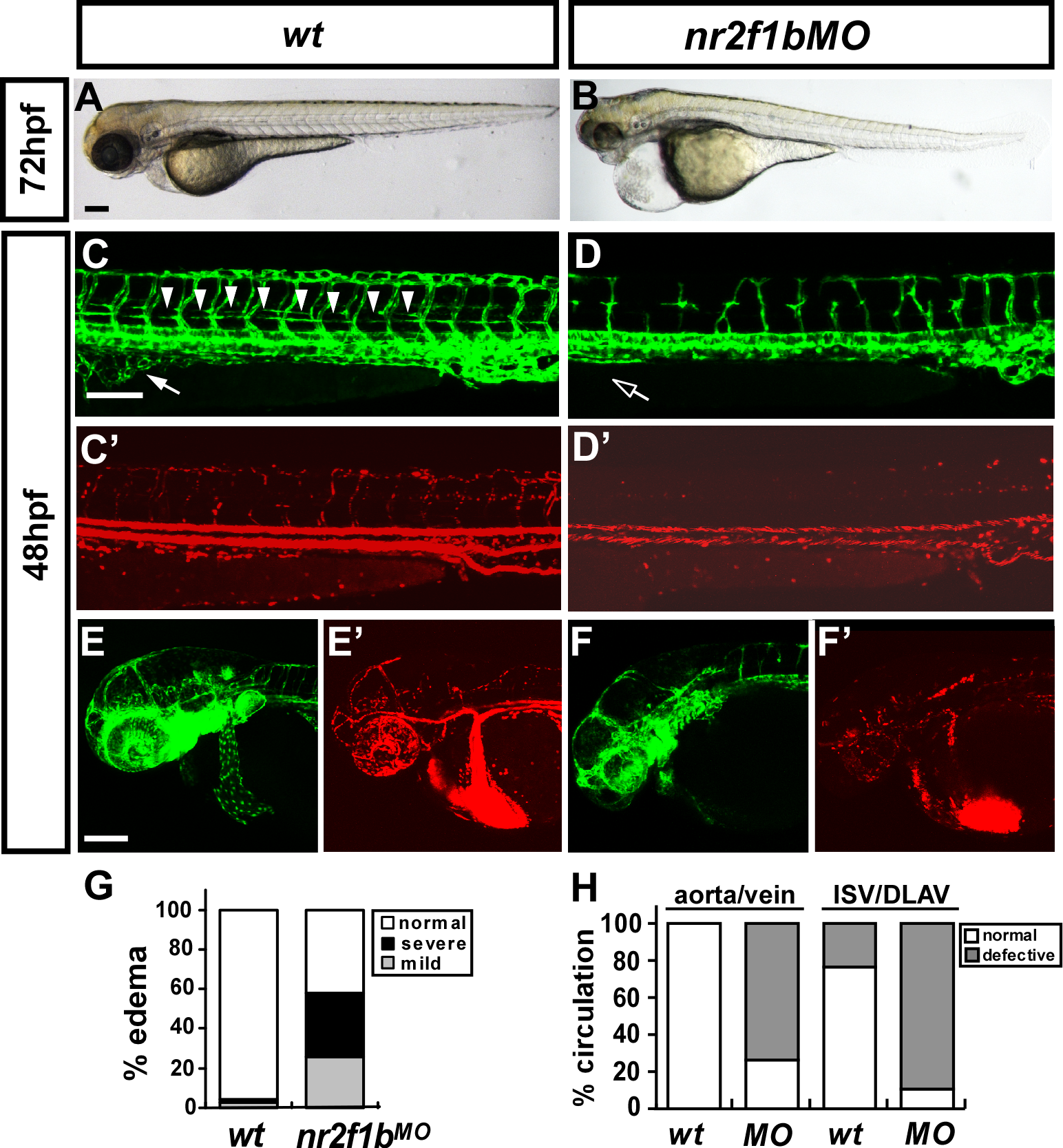


**Figure S3. Loss of *nr2f1b* results in pericardial edema, absent parachordal vessels, subintestinal vessels (SIV) mispattern and circulation defects.**

(A,B) *nr2f1b*MO injected embryos showed severe pericardial edema at 72hpf compared to wt, in addition, there is an obvious smaller eyes in *nr2f1b* morphants. Quantitatively, 60% of *nr2f1b* morphants (n=43) with mild to severe pericardial edema compared to wt (n=49) as shown in (G). (C-F, C’-F’) *nr2f1b* ^i1e2^ MO was injected into transgenic *Tg(fli:eGFP^y1^;gata1:dsRed^sd2^)* embryos with GFP-labeled endothelial cells (**C,D,E,F**) and dsRed-labeled blood cells(**C’,D’,E’,F’**). *nr2f1b* morphants show mispatterned ISV, DLAV and SIV (*arrow*), and absent parachordal vessels (*arrowhead*) (C and D) and circulation defect at the ISV and DLAV and slow to lose axial circulation of the aorta and vein (**D’**) in the trunk region at 48hpf as compared to wild-type (**C’**). Vasculature and circulation defects are also observed in the head region of *nr2f1b* morphants (F and **F’**) as compared to wt (E and **E’**). Quantification of the number of embryos exhibiting trunk vessel circulation defects in wt (n=8) and *nr2f1b* morphants (n=25) as shown in (**H**). The scale bars in C-F and C’-F’ represent 100 μm and in A-B are 500 μm.
